# Maximizing Efficiency in Energy Trading Operations through IoT-Integrated Digital Twins

**DOI:** 10.3390/s23249656

**Published:** 2023-12-06

**Authors:** Faiza Qayyum, Reem Alkanhel, Ammar Muthanna

**Affiliations:** 1 Department of Computer Engineering, Jeju National University, Jeju-si 63243, Republic of Korea; 2 Department of Information Technology, College of Computer and Information Sciences, Princess Nourah bint Abdulrahman University, Riyadh 11671, Saudi Arabia; 3 Department of Telecommunication Systems, Peoples’ Friendship University of Russia (RUDN University), 6 Miklukho-Maklaya, 117198 Moscow, Russia; muthanna.asa@sut.ru

**Keywords:** Internet of Things, complex problem solving, critical IoT systems, nano-grid, optimization, task modeling, task orchestration

## Abstract

The Internet of Things (IoT) has brought about significant transformations in multiple sectors, including healthcare and navigation systems, by offering essential functionalities crucial for their operations. Nevertheless, there is ongoing debate surrounding the unexplored possibilities of the IoT within the energy industry. The requirement to better the performance of distributed energy systems necessitates transitioning from traditional mission-critical electric smart grid systems to digital twin-based IoT frameworks. Energy storage systems (ESSs) used within nano-grids have the potential to enhance energy utilization, fortify resilience, and promote sustainable practices by effectively storing surplus energy. The present study introduces a conceptual framework consisting of two fundamental modules: (1) Power optimization of energy storage systems (ESSs) in peer-to-peer (P2P) energy trading. (2) Task orchestration in IoT-enabled environments using digital twin technology. The optimization of energy storage systems (ESSs) aims to effectively manage surplus ESS energy by employing particle swarm optimization (PSO) techniques. This approach is designed to fulfill the energy needs of the ESS itself as well as meet the specific requirements of participating nano-grids. The primary objective of the IoT task orchestration system, which is based on the concept of digital twins, is to enhance the process of peer-to-peer nano-grid energy trading. This is achieved by integrating virtual control mechanisms through orchestration technology combining task generation, device virtualization, task mapping, task scheduling, and task allocation and deployment. The nano-grid energy trading system’s architecture utilizes IoT sensors and Raspberry Pi-based edge technology to enable virtual operation. The evaluation of the proposed study is carried out through the examination of a simulated dataset derived from nano-grid dwellings. This research analyzes the efficacy of optimization approaches in mitigating energy trading costs and optimizing power utilization in energy storage systems (ESSs). The coordination of IoT devices is crucial in improving the system’s overall efficiency.

## 1. Introduction

**Figure 1:**
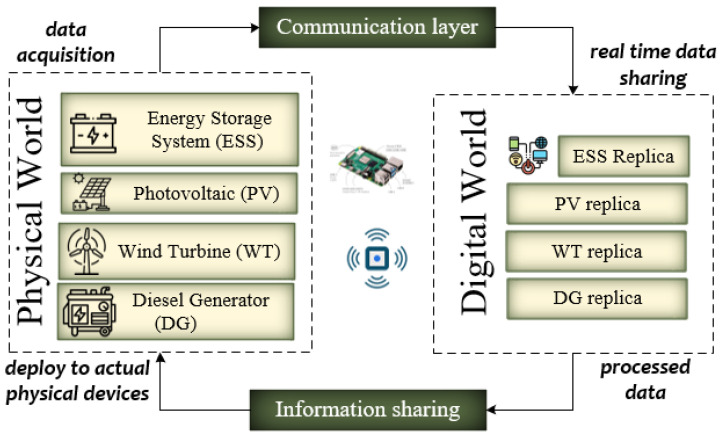
Conceptual overview of digital twin technology.

The Internet of Things (IoT) has resulted in notable transformations across multiple domains, encompassing smart residences, urban settings, and healthcare implementation [1,2,3,4]. One of the key contributors to the advancement of Internet of Things (IoT) technology was Ashton Kevin, who argued for the notion of digitally modifying the physical world to enable intelligent operations (Ashton [5,6,7]). The Internet of Things (IoT) is a networked framework that facilitates connections between physical objects, such as sensors, software, or similar technologies. This framework enables efficient communication between these devices and individuals over the Internet. The devices encompass a wide spectrum, including advanced industrial gear and everyday household items [8]. Based on empirical research about the substantial expansion of the Internet of Things (IoT) framework, projections indicate that the quantity of IoT-enabled gadgets is anticipated to surpass 25.4 billion by 2030. It is anticipated that roughly 33% of the overall amount will be allocated specifically for mission-critical applications [9]. Mission-critical systems encompass various applications, such as real-time navigation, healthcare, and energy power [4,5,6,7,10,11]. The inception of microgrids as a concept can be attributed to R.H. Lasseter in 2002. Microgrids are decentralized distribution systems that function at reduced voltage levels and significantly emphasize promoting environmental sustainability. The technologies are specifically engineered to oversee distributed generation systems efficiently [12]. A less complex version of a microgrid, a nano-grid, is often characterized by a load that serves a single family [13]. The scientific community has focused on overcoming shortcomings in minimizing power loss in DC microgrids by employing both the Lagrange multiplier and the distributed gradient algorithm [14,15]. A nano-grid is widely recognized as an independent system that oversees operations, regulates voltage, and ensures reliability. Generally, the system comprises at least one gateway, establishing an external connection [16]. Integrating a nano-grid system within a healthcare clinic, comprising linked nano-grids that provide peer-to-peer (P2P) exchange of environmentally sustainable energy, can be considered a pivotal and significant application. The term “peer-to-peer (P2P) energy operation” refers to the decentralized management and operation of energy generation, consumption, and trade inside a nano-grid system. In the context of these conditions, it is imperative to guarantee a consistent and uninterrupted provision of energy. IoT technology can enhance the efficiency of crucial mission-critical power structures, similar to the indispensable systems in healthcare [4]. This can be achieved by optimizing fundamental aspects of energy operations. An exemplification of such a system is elucidated in [17], wherein the utilization of IoT task orchestration is employed to enhance the efficacy of the nano-grid energy management system. Task orchestration effectively coordinates and automates various jobs and processes by replicating virtual objects. So far, there needs to be more research that explores the utilization of Internet of Things (IoT) technology in the energy sector, specifically in the context of smart grid energy operations, such as energy trading and management. This contrasts the healthcare domain, where IoT technology has been extensively studied and implemented. The utilization of Internet of Things (IoT) technology has the potential to significantly improve the functionality of diverse applications related to the management of smart grids. Employing Internet of Things (IoT) task orchestration within nano-grid energy trading can augment energy transactions’ effectiveness, dependability, and mechanization. This implementation also facilitates the instantaneous monitoring, regulation, and enhancement of the nano-grid system. As previously stated, integrating IoT technology has led to substantial breakthroughs in mission-critical systems within the healthcare and navigation sectors. This integration has replaced manual operations with fully automated systems that utilize edge-based IoT technologies. Nevertheless, numerous research studies have been conducted to explore the utilization of Internet of Things (IoT) technology in energy operations, particularly in smart grid architecture, with a specific emphasis on microgrid and nano-grid energy operations. According to several research, it has been shown that the building sector is accountable for approximately 40% of worldwide energy use. By strategically incorporating IoT orchestration into nano-grid energy trading systems, managing the entire framework orchestrated becomes feasible, improving overall efficiency [5,6]. The utilization of Internet of Things (IoT) technology has the potential to enhance energy systems’ capabilities greatly. The effectiveness of orchestration in mission-critical systems has been demonstrated in its ability to facilitate autonomous process management [18]. The term “orchestration” pertains to the automated administration and configuration of system components facilitated by the Internet of Things (IoT), specifically in scenarios that need immediate responsiveness [5,6]. However, orchestration is widely utilized in other fields, like healthcare and power systems, which still necessitate orchestration to leverage its scalability through automated energy operations. The literature has previously discussed service-level orchestration, but there needs to be more focus on implementing task-level orchestration [5]. A digital twin is a virtual representation of a real-world object, system, or process created by collecting and integrating data from various sources, such as sensors, simulations, and historical records, as shown in Figure 1. It is a dynamic and interactive model mimicking its physical counterpart’s behavior, status, and characteristics. Digital twins are used for analysis, monitoring, prediction, and optimization, enabling insights into real-world performance, maintenance, and potential outcomes, particularly in manufacturing, healthcare, and infrastructure management.

The critical analysis of contemporary state-of-the-art methods revealed that the energy sector has seen limited exploration of the Internet of Things (IoT) technology, specifically in smart grid operations, like energy power management in distributed generation-based systems. While there is extensive research on IoT applications in healthcare, the energy domain needs a comparable depth of exploration. Furthermore, there needs to be more research focusing on digital twin-based IoT task orchestration within energy systems, particularly in nano-grids. The aforementioned issues have motivated the following key contributions:

- This study introduces a novel IoT-enabled digital twin perspective on nano-grid energy system orchestration by proposing the inclusion of task-level orchestration, a feature currently lacking in the existing research.
- The incorporation of digital twins focuses on the scalability of the nano-grid system by replicating physical resources through virtual objects.
- To optimize the energy operations of energy storage systems (ESSs) before peer-to-peer (P2P) trading, the proposed study implements PSO-enabled ESS power optimization.
- The application of particle swarm optimization (PSO) can enhance the efficiency of the energy storage system (ESS), facilitating the optimal management of surplus energy.
- By conducting a comprehensive evaluation of the system’s performance through a case study and comparison analysis, this study aims to contribute significant insights to the current body of knowledge.
- The evaluation of the virtualization module encompasses an examination of round-trip time analysis (RTT), latency analysis (LA), and response time (RT).
- A comparative study analyzes the system’s contribution to the existing literature body, highlighting its unique characteristics and achievements.

## 2. Related Work

This section provides an overview of the current research on orchestration architectures based on IoT in the context of smart grid and healthcare applications. Recognizing the predictable attributes inherent in mission-critical systems, such as the real-time power allocation to smart grid infrastructure, holds substantial importance [19]. The successful implementation of the Internet of Things (IoT) enabled applications crucial for mission-critical operations, which necessitate the ability to respond to events in real-world situations promptly. The emergence of the Internet of Things (IoT) has greatly increased the prevalence of devices equipped with intelligent sensors [18]. Consequently, this development has enhanced performance in various fields, such as smart cities [20,21] and enterprise systems [22]. The progress made in the Internet of Things (IoT) field has created new avenues for creating and enhancing highly effective IoT applications. In service-oriented architecture, orchestration has gained significant importance in design [23,24]. Its primary objective is to automate the complete execution process. There has been considerable scholarly interest in orchestration, specifically focusing on its use in service-oriented architecture (SOA) [25,26].

IoT has become an essential element in both conventional and modern applications. A study by [27] introduced the concept of “IoT ProSe”, incorporating task orchestration within mobile nodes. Additional methodologies, such as ProFun [28] and Makesense [29], were developed to enhance task execution efficiency. It is important to note that these methodologies primarily emphasized the dynamic features of orchestration rather than its autonomous behavior. The healthcare industry and other industries successfully utilized the potential of IoT technology by integrating orchestration [4]. In reference [6], a proposed architecture known as MDMT-MOA was presented for IoT enterprise designs, primarily focusing on orchestration. In a separate investigation, a scholarly article [30] presented the notion of cyber–physical systems as a service (CPSaaS) to achieve efficient task coordination in smart urban environments. The paper [31] introduced a mission-critical architecture designed to detect mountain fires, utilizing IoT task orchestration and integrating essential modules, including microservices and predictive analysis. Various scholarly investigations, such as healthcare research [32,33], utilized sensors to identify patient motion in IoT health monitoring systems. The study by [33] also introduced a healthcare monitoring architecture that utilized cloud and fog-based computing systems to facilitate effective healthcare data administration.

In [4], a research study introduced the concept of IoT task orchestration to monitor patient health. This study also proposed using an optimized scheduling technique to execute healthcare jobs efficiently. The study employed a hierarchical methodology for the coordination of tasks, involving the stages of task generation, task mapping, task scheduling, task allocation, and task deployment. The aforementioned methodology resulted in notable enhancements in response time and diminished latency. Furthermore, recent scholarly investigations on orchestration within the domain emphasized the automation of providing services. Several research projects were conducted to implement task-level orchestration, providing various benefits like increased flexibility, robustness, and dynamic management of the overall system execution [5,6,7]. Scheduling tasks was of great importance in orchestrating tasks within the Internet of Things (IoT) framework, and optimizing this process could substantially impact the system’s overall performance.

Recently, various efforts were made in terms of energy efficiency. The research [34] focused on the topic of secrecy of energy efficient hybrid beamforming in integrated satellite–terrestrial networks. The objective was to optimize the trade-off between secrecy and energy efficiency, taking into account the presence of imperfect angles of departure. This study presented two resilient beamforming strategies for both single and multiple earth stations, showcasing their efficacy through simulations utilizing realistic channel models. Similarly, the study [35] centered on the augmentation of security and energy efficiency in multibeam satellite systems through the optimization of system secrecy and energy efficiency while adhering to a total power constraint. The nonconvex issue was decomposed into subproblems using an alternating optimization strategy, which incorporated the signal-to-leakage-plus-noise ratio and consecutive convex approximation techniques. The simulations provided evidence of the higher performance of the proposed strategy compared to benchmark alternatives in terms of security and energy efficiency. Another study [36] examined the issues associated with IoT connectivity and proposed a solution by exploring the concept of rate-splitting multiple access (RSMA) within a satellite and aerial-integrated network (SAIN). This study aimed to enhance multicast communication for Internet of Things devices (IoTDs) in a content delivery context, with a specific focus on mitigating interference and improving spectral efficiency. The RSMA approach, as suggested, was guided by an iterative optimization algorithm. This method significantly improved the sum rate of the system and reduced mutual interference. It was shown to outperform benchmark schemes in the context of IoT applications. This study centered on enhancing communication efficiency inside a satellite–terrestrial-integrated network (STIN) by utilizing non-orthogonal multiple access (NOMA). The study [37] proposed a novel approach combining beamforming and power allocation techniques to optimize the overall data transmission rate while ensuring adherence to quality-of-service requirements and power limitations for both satellite and cellular users. The suggested methodology, incorporating a unique user pairing scheme and utilizing an iterative penalty function-based beamforming technique, demonstrated superior performance compared to current approaches. This advancement held promising advantages for the field of Internet of Things (IoT) communication within integrated networks. The existing studies are delineated in Table 1.

Based on a critical analysis of the existing studies, the proposed study aims to leverage IoT technology and digital twin-based methods to transition traditional smart grids into IoT-based frameworks for more efficient energy resource sharing. It covers advantages like enhanced energy trading efficiency and reduced expenses through optimization techniques. The study addresses the untapped potential of IoT in the energy sector, particularly in improving energy resource sharing and overall system performance.

## 3. Proposed Architecture

**Figure 2:**
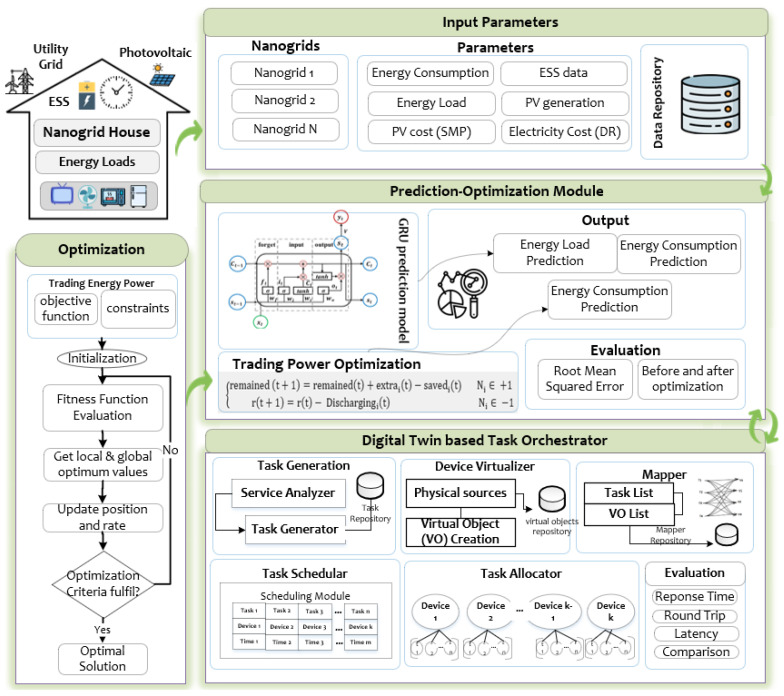
Architecture details for nano-grid energy management via Internet of Things (IoT)-enabled digital twin concept.

This section establishes a system for energy trading within nano-grids using a digital twin and task coordination based on the Internet of Things (IoT). The primary concept revolves around using sensors and the Internet of Things (IoT) to effectively oversee the energy trading procedure within nano-grids, diminishing the expenses associated with the upkeep of tangible resources. The proposed methodology encompasses two primary components: the enhancement of energy storage systems for residential units within the nano-grid that engage in peer-to-peer energy exchange and the virtual execution of the complete energy trading procedure through the Internet of Things (IoT)-based task coordination to reduce expenses associated with tangible resources, such as solar panels and energy storage batteries. The architectural representation of this system is depicted in Figure 2. The initial step involves collecting input data about each module within the nano-grid. This includes information on energy consumption, energy storage system (ESS) data, energy load, energy generation from solar panels (photovoltaic or PV), electricity costs, and relevant photovoltaic details. The aforementioned data are sent to the prediction module, which employs a prediction technique based on gated recurrent units (GRUs) to anticipate energy load, photovoltaic (PV) generation, and energy consumption. Before producing predictions, it is imperative to analyze the input data thoroughly. Subsequently, a subset of these forecasts is utilized within the energy trading module to effectively optimize the energy costs.

The proposed design incorporates the concept of a digital twin, consisting of two primary components: the “virtual space” and the “physical space”. Within virtual environments, the preliminary stages of task management encompass several activities, including creating tasks, generating virtual representations of devices, formulating task schedules, and allocating those tasks. In contrast, the physical space encompasses the virtual resources utilized for the efficient execution of tasks.

### 3.1. GRU Prediction Module

This refers to a system component that employs a specialized type of neural network known as gated recurrent units (GRUs) to perform predictive tasks. The GRUs are specialized components within the network that demonstrate proficiency in capturing patterns within sequential data. As a result, these components enhance the module’s ability to generate precise forecasts or guesses.

### 3.2. ESS Power Management for Optimal Energy Trading

Nano-grids connect with the primary utility grid through specialized converters, namely bi-directional direct current to alternating current (BD-DC-AC) converters, to maintain an equilibrium in energy distribution. In surplus energy inside a nano-grid system, the converter is designed to transition into a mode whereby excess energy is redirected and transmitted back to the primary grid. Conversely, when the energy supply in a nano-grid is insufficient, the converter draws energy from the primary grid. The definitions of the acronyms employed in the ideal energy storage system (ESS) for nano-grid residences are located in Table 2.

(1) $$H=\{H_{1},H_{2},H_{3},\ldots ,H_{k}\}$$

The symbol ±1 represents the energy exchange through buying and selling. For instance, assigning a value of ’+1’ to a nano-grid indicates that the nano-grid possesses a surplus of energy that can be exchanged with other nano-grids in the peer-to-peer (P2P) network. Alternatively, when the allocated value is ’−1’, it signifies that the nano-grid is deficient in energy to fulfill its energy demands. Consequently, it must obtain energy from other interconnected nano-grids (with priority given to them) or the utility grid. The term “battery” pertains to the assemblage of energy storage technologies implemented within every nano-grid. To illustrate,

(2) $$battery=\{battery_{1},battery_{2},battery_{3},\ldots ,battery_{k}\}$$

In the present scenario, the variable *i* represents the aggregate quantity of energy storage systems (ESSs), tantamount to the number of residences outfitted with a nano-grid. Let *t* represent a specific time interval, whereas *L* represents the load demand. Hence, the variable ${\mathrm{EL}}_{k}\left(t\right)$ denotes the load demand for the *i*th nano-grid at a given time *t*.

The variable ${\mathrm{photovoltaic}}_{k}\left(t\right)$ denotes the power produced by the photovoltaic (PV) system in the *k*th nano-grid at a given time *t*. This power can be utilized for either charging ${\mathrm{battery}}_{k}$ or fulfilling the energy demand ${\mathrm{EL}}_{k}\left(t\right)$. Furthermore, any surplus energy generated by the photovoltaic (PV) system has the potential to be traded with other nano-grids experiencing a shortage. The energy produced by the photovoltaic (PV) system to meet the power requirements of its nano-grid is commonly known as self-supplied energy. This term is defined as follows:

(3) $${\mathrm{extra}}_{k}\left(t\right)=\min\left\{{\mathrm{EL}}_{k}\left(t\right)\times {\mathrm{photovoltaic}}_{k}\left(t\right)\right\}$$

The energy storage system (ESS) unit consists of batteries used to store energy produced by the photovoltaic (PV) system and a bi-directional DC-DC converter. The energy in the battery at a given time interval, denoted ${\mathrm{remained}}_{k}\left(t\right)$, is commonly referred to as leftover energy.

The following scenarios are considered for peer-to-peer (P2P) energy sharing/trading:

1. In a situation when the trading function yields a positive value of +1, priority will be assigned to the exporting of energy through ${\mathrm{photovoltaic}}_{k}\left(t\right)$. This implies that any surplus energy the photovoltaic system produces at time *t* will be used for trading. In the event of a lack of further energy, the stored energy within a battery(t) at time *t* is employed for trading.
2. In instances when the trade function is assigned a value of −1, it is important to note that the acquired energy is exclusively applicable for immediate power supply purposes and cannot be kept within the ${\mathrm{battery}}_{k}$ system.

The energy that is left over following energy trading over the specified period *t* is commonly known as:

(4) $$\mathrm{remained}=\{{\mathrm{remained}}_{1},{\mathrm{remained}}_{2},{\mathrm{remained}}_{3},\ldots ,{\mathrm{remained}}_{k}\}$$

The term ’remained’ denotes the amount of energy still present in the battery of the *k* nano-grid at a given time, denoted as *t*. The term ’remained’ is subject to a constraint that is determined by the effective system size (ESS) capability. The equation presented below represents the constraint:

(5) $${\mathrm{remained}}_{\min,k}\leq r_{k}\leq {\mathrm{remained}}_{\max,k}$$

The variable ${\mathrm{remained}}_{\max,k}$ denotes the maximum energy storage system (ESS) capacity in kilowatt-hour (kWh) for the *i* nano-grid. On the other hand, $r_{\max,k}$ indicates the minimum ESS energy determined by the depth of discharge (DoD) to avoid excessive discharge.

The energy transfer occurring among households (i.e., nano-grid) is denoted as ${\mathrm{extra}}_{k}\left(t\right)$, representing the amount of energy exchanged by the *k*th nano-grid at a specific time *t*. The quantity of ${\mathrm{extra}}_{k}\left(t\right)$ will function as a conclusive determinant, simultaneously reflecting the magnitude of surplus energy within the nano-grid. If the total energy shared surpasses zero, the trade function will receive a numerical value of +1. Moreover, the additional ${\mathrm{extra}}_{k}\left(t\right)$ value is also beneficial in assessing the quantity of extra energy accessible within the nano-grid. If the total energy surpasses zero, the trade function will become +1; otherwise, it will be assigned a value of −1.

The subsequent limitations are imposed on the practice of energy sharing. The symbol $\sum_{k}{\mathrm{extra}}_{k}\left(t\right)$ represents the residual energy in *N*. The energy exchanged within the nano-grid at a given time *t*, denoted as $\sum_{k}{\mathrm{extra}}_{k}\left(t\right)$, must fall within the interval of 0, and the maximum allowable energy that can be shared during that specific time. The surplus energy solar power generates is commonly denoted as $\sum_{k}{\mathrm{photovoltaic}}_{k}\left(t\right)$. The subsequent function defines the value of energy sharing.

(6) $${\mathrm{extra}}_{k}\left(t\right)=\left\{\begin{array}{cc} \min\left({\mathrm{photovoltaic}}_{k}\left(t\right)-{\mathrm{EL}}_{k}\left(t\right),A_{\max,i}\right) & \mathrm{if}\;H_{i}\in +1\\ 0 & \mathrm{if}\;H_{i}\in -1 \end{array}\right.$$

The determination of the value of ${\mathrm{extra}}_{k}\left(t\right)$ is contingent upon two factors: (1) the maximum energy capacity of ${\mathrm{battery}}_{i}$ at time *t*, as indicated by the rated power of the DC-DC converter, and (2) the maximum remaining capacity of ${\mathrm{remained}}_{\max,k}$. The phrase ${\mathrm{extra}}_{k}\left(t\right)$ refers to the released or discharged energy. The battery, denoted as ${\mathrm{battery}}_{k}$, serves as the energy source for the *k*th nano-grid, as represented by the given expression.

(7) $${\mathrm{dis}}_{k}\left(t\right)=\left\{\begin{array}{cc} 0 & \mathrm{if}\;H_{i}\in +1\\ \min({\mathrm{EL}}_{k}\left(t\right)-{\mathrm{photovoltaic}}_{k}\left(t\right),{\mathrm{EL}}_{\max,k}) & \mathrm{if}\;H_{i}\in -1 \end{array}\right.$$

The greatest amount of energy that can be released from $e_{i}$ during a given period, written as ${\mathrm{dis}}_{\max,i}$, is determined based on the remaining energy in the energy storage system (ESS) and the power capacity of the bidirectional DC-DC converter. The determination of energy flow is made based on the utilization of the numerical values of +1 and −1, which are outlined as follows:

(8) $$\mathrm{remained}\left(t+1\right)=\left\{\begin{array}{cc} \mathrm{remained}\left(t\right)+{\mathrm{extra}}_{k}\left(t\right)-{\mathrm{saved}}_{k}\left(t\right) & \mathrm{if}\;H_{k}\in +1\\ \mathrm{remained}\left(t\right)-{\mathrm{rel}}_{k}\left(t\right) & \mathrm{if}\;H_{k}\in -1 \end{array}\right.$$

To obtain energy (+1), the variable $e_{i}\left(t\right)$ must adhere to two requirements. If the value of *H* is equivalent to 1 and the surplus energy of the *k*th nano-grid during time interval *t* is not greater than the energy stored in the battery at time interval *t* (i.e., ${\mathrm{extra}}_{k}\left(t\right)\leq {\mathrm{battery}}_{k}\left(t\right)$), then the discharging mode is initiated. Furthermore, the surplus power of the nano-grid at time (k+1) can be determined by subtracting the surplus energy of the *i*th nano-grid house during time interval *t*, denoted as ${\mathrm{extra}}_{k}\left(t\right)$, from the energy stored in the *k*th nano-grid at timestamp *t*, represented as ${\mathrm{battery}}_{k}\left(t\right)$. Alternatively, if the surplus energy of the *m*th nano-grid at timestamp *t* is lower than the energy stored in the *k*th nano-grid’s energy storage system (ESS), the charging mode is activated. Consequently, the excess energy for the (i+1)th nano-grid during the time interval *t* is determined by subtracting the surplus energy of the *i*th nano-grid house from the energy stored in the ESS of the *i*th nano-grid at time *t*, denoted as ${\mathrm{extra}}_{k}\left(t\right)-{\mathrm{battery}}_{k}\left(t\right)$.

The operational limitations for entity $e_{i}$ throughout the charging process exhibit variations when the value of NG is −1 instead of +1. The aforementioned limits are contingent upon the energy usage and availability of each nano-grid. The term $\mathrm{traded}\;\mathrm{energy}\;{\mathrm{sold}}_{k}\left(t\right)$ is defined as:

(9) $$f\left(x_{i}\right)={\mathrm{sold}}_{k}\left(t\right):\max\left({\mathrm{remained}}_{k}\left(t\right)-{\mathrm{remained}}_{\min,k}+{\mathrm{extra}}_{k}\left(t\right),0\right)$$

The given equation is subject to the following limitations:

$$-{\mathrm{remained}}_{k}\left(t\right)\leq {\mathrm{battery}}_{k}\left(t\right)\leq 0$$

Moreover, it is imperative to ensure that the exchange of energy between nano-grids with contrasting values, wherein one nano-grid possesses a negative value while the other has a positive value, does not exceed the quantity of energy the latter nano-grid has at its disposal for trading purposes.

For a detailed explanation, we also provide the methodology of the proposed study in the form of a detailed algorithm shown in Algorithm 1.
**Algorithm 1** Optimal Energy Trading using PSO 1:**Input:** Define the following variables and sets: 2:Set of participating nano-grid houses: $H=\{H_{1},H_{2},H_{3},\ldots ,H_{k}\}$ 3:Energy Storage System (ESS) installed in each house: $battery=\{battery_{1},battery_{2},battery_{3},\ldots ,battery_{k}\}$ 4:Time interval: *t* 5:Load demand: $EL_{k}\left(t\right)$ for the *k*th nano-grid 6:Photovoltaic power: $photovoltaic_{k}\left(t\right)$ for the *k*th nano-grid 7:Self-supplied energy: $extra_{k}\left(t\right)=\min\left\{EL_{k}\left(t\right)\cdot photovoltaic_{k}\left(t\right)\right\}$ 8:Remaining energy in battery: $remained_{k}\left(t\right)$ 9:Energy load: $rel_{k}\left(t\right)$10:Energy saved in ESS: $saved_{k}\left(t\right)$11:Sold energy: $sold_{k}\left(t\right)$12:**Particle Swarm Optimization:**13:Initialize particle positions $X_{i}$ and velocities $V_{i}$ for i=1 to *N* using problem-specific initialization strategies;14:**while** stopping criterion not met **do**15:      **for** i=1 to *N* **do**16:         **if** Trade function yields +1 **then**17:             Update ${\mathrm{remained}}_{k}\left(t+1\right)={\mathrm{remained}}_{k}\left(t\right)+{\mathrm{extra}}_{k}\left(t\right)-{\mathrm{saved}}_{k}\left(t\right)$18:         **else if** Trade function yields −1 **then**19:             Update ${\mathrm{remained}}_{k}\left(t+1\right)={\mathrm{remained}}_{k}\left(t\right)-{\mathrm{rel}}_{k}\left(t\right)$20:         **end if**21:     **end for**22:     Calculate ${\mathrm{extra}}_{k}\left(t\right)$ representing energy exchanged by the *k*th nano-grid at time *t*23:     Enforce $\sum_{k}{\mathrm{extra}}_{k}\left(t\right)\in \left[0,\max\;\mathrm{allowable}\;\mathrm{energy}\right]$24:     Calculate $\sum_{k}{\mathrm{photovoltaic}}_{k}\left(t\right)$25:     Calculate ${\mathrm{extra}}_{k}\left(t\right)$ using Equation (6)26:     Calculate ${\mathrm{rel}}_{k}\left(t\right)$ using Equation (7)27:     Enforce $\sum_{k}{\mathrm{extra}}_{k}\left(t\right)\in \left[0,\max\;\mathrm{allowable}\;\mathrm{energy}\right]$28:     **for** k=1 to *k* **do**29:           Update ${\mathrm{remained}}_{k}\left(t+1\right)$ according to Equation (8)30:           Calculate ${\mathrm{sold}}_{k}\left(t\right)$ using Equation (9)31:           Enforce $-{\mathrm{remained}}_{k}\left(t\right)\leq {\mathrm{battery}}_{k}\left(t\right)\leq 0$ according to Equation (10)32:     **end for**33:     **Particle Swarm Optimization Update:**34:     **for** i=1 to *N* **do**35:           Evaluate the objective function $f(X_{i})$ using Equation (9)36:           **if** $f(X_{i})$ is better than the personal best of particle *i* **then**37:              Update personal best: $P_{\mathrm{best},i}=X_{i}$38:           **end if**39:     **end for**40:     Find the global best: $G_{\mathrm{best}}=\arg\min f\left(P_{\mathrm{best},i}\right)$ over all particles41:     **for** i=1 to *N* **do**42:           Update velocity: $V_{i}=\omega V_{i}+c_{1}r_{1}\left(P_{\mathrm{best},i}-X_{i}\right)+c_{2}r_{2}\left(G_{\mathrm{best}}-X_{i}\right)$43:           Update position: $X_{i}=X_{i}+V_{i}$44:           Enforce constraints from Equation (10): $-{\mathrm{remained}}_{k}\left(t\right)\leq {\mathrm{battery}}_{k}\left(t\right)\leq 0$45:     **end for**46:**end while**47:**Output:** Optimal solution $G_{\mathrm{best}}$

### 3.3. IoT-Enabled Task Orchestration-Based Energy Trading Operation

**Figure 3:**
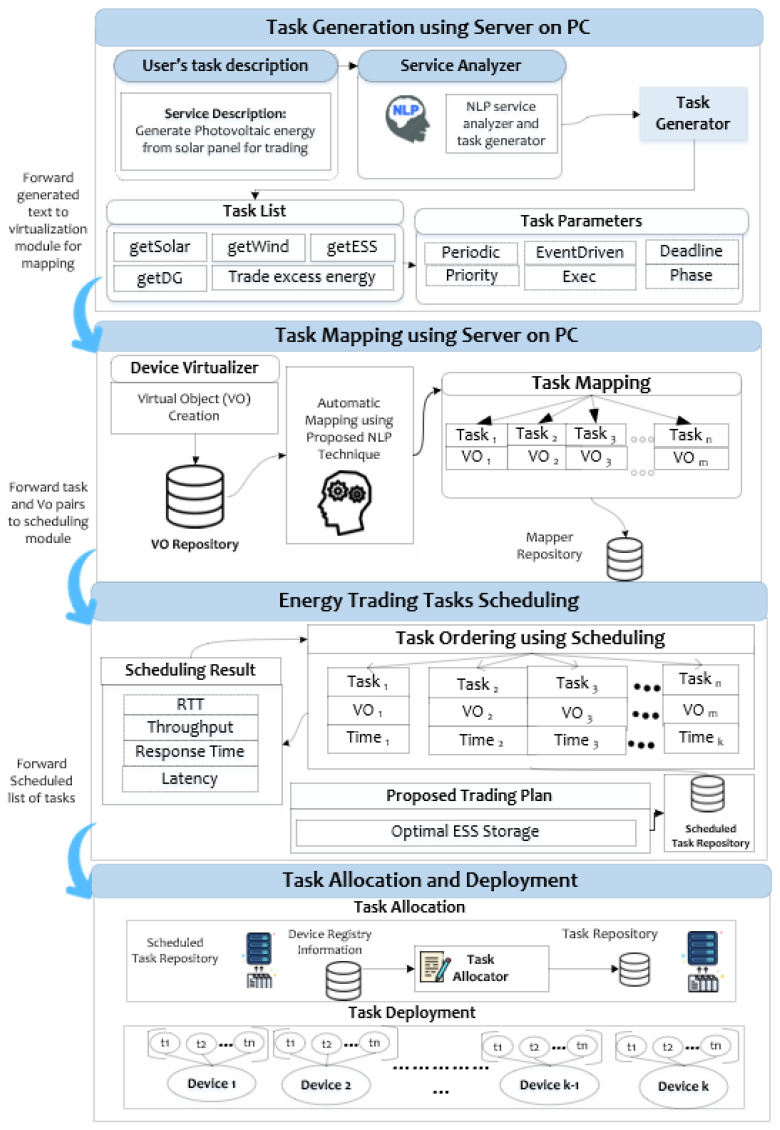
Digital twin-based Internet of Things (IoT) task orchestration layered viewer for optimal energy trading.

This study employs an Internet of Things (IoT) framework for task orchestration, utilizing digital twin technology to replicate physical resources and virtualize them within the P2P nano-grid, facilitating energy trading. The methodology involves five main steps of the digital twin, including task generation, virtual object creation, task mapping, task scheduling, task allocation, and deployment through natural language processing (NLP) and the creation of virtual objects. Task scheduling prioritizes tasks based on priority level and deadline, optimizing the sequence for IoT devices. The task allocator assigns tasks to specific devices, enhancing temporal efficiencies, and deployment concludes the process. Our proposed study covers the integration of digital twin-based IoT orchestration, task automation, and efficient energy resource management within the nano-grid context, offering a comprehensive solution for enhanced energy trading and system performance. Figure 3 shows the conceptual overview of digital twin concepts in the form of task orchestration. The layered view starts with task generation, which is generated based on a user-provided description, followed by task mapping using a server on a PC, energy trading tasks scheduling, and the final step of task allocation and deployment.

**Figure 4:**
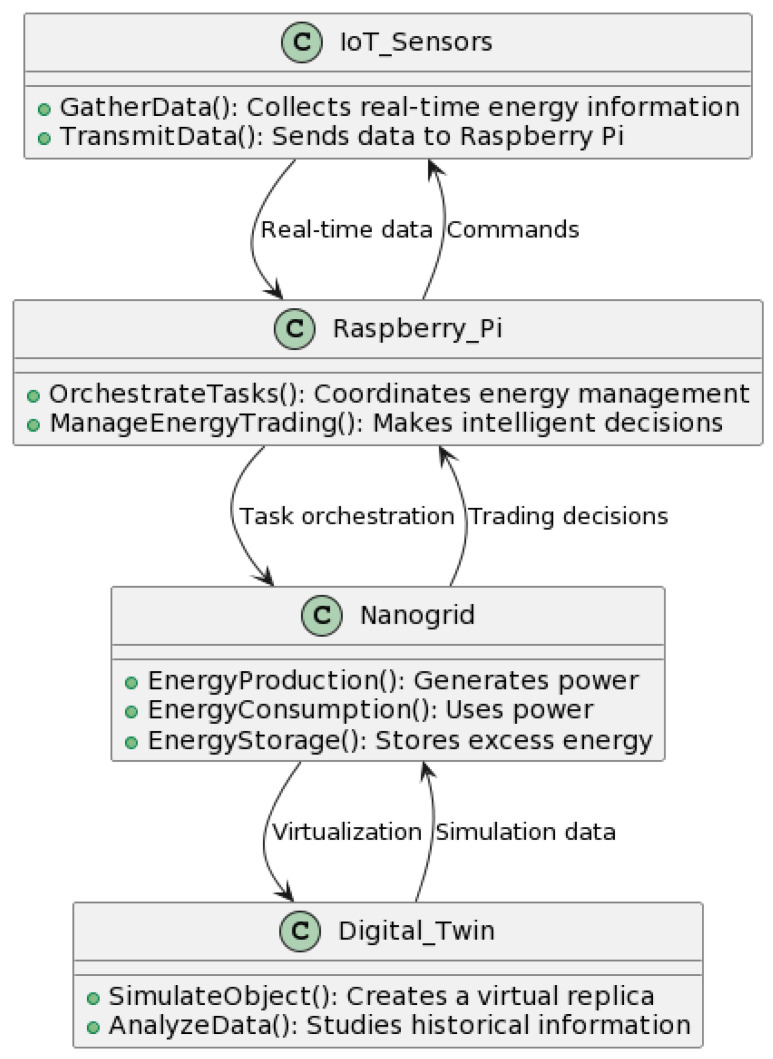
Overview of Internet of Things (IoT) sensors and Raspberry Pi in proposed IoT-orchestrated Network.

The utilization of IoT sensors and Raspberry Pi devices plays a crucial part in the task orchestration of peer-to-peer energy trading within the virtualization process. Internet of Things (IoT) sensors are strategically positioned to collect real-time data pertaining to the production, consumption, and storage of energy inside a nano-grid. The data are subsequently transferred to a central orchestrator, which is most likely built on a Raspberry Pi. This orchestrator utilizes task orchestration principles to dynamically oversee and enhance the process of energy trading between multiple participants. By utilizing the data obtained from Internet of Things (IoT) sensors and harnessing the computing capacities of Raspberry Pi, the system is able to make well-informed decisions, effectively coordinating the process of peer-to-peer energy trading in a timely manner. This approach improves the efficiency of the virtualized energy trading system by combining insights from the Internet of Things (IoT) with the automated job management capabilities of the Raspberry Pi platform. The diagram in Figure 4 depicts a framework for facilitating peer-to-peer energy trading through Internet of Things (IoT) sensors, a Raspberry Pi, nano-grid technology, and a digital twin. As shown, Internet of Things (IoT) sensors are utilized to collect real-time data about energy consumption, which is subsequently transferred to the Raspberry Pi. The Raspberry Pi is the central controller for task coordination and energy trading within the nano-grid system. The nano-grid, which serves as a tangible energy infrastructure, interacts with a digital twin for virtualization. The concept of the digital twin involves the simulation of physical entities and the analysis of past data, hence facilitating the development of a comprehensive framework for optimizing energy management and trading processes.

### 3.4. Evaluation

This section explains the evaluation measures utilized to assess the potential of each module within the proposed energy trading architecture.

#### 3.4.1. Root Mean Square Error

The determination of prediction outcomes for energy consumption, load, and PV generation prediction is based on using RMSE square values. These values are also compared with the findings obtained by the BD-LSTM model. The equation below represents the formula for calculating the root mean square error (RMSE) value. In this equation, *R* represents the real or actual values, *P* represents the values predicted by the GRU model, and *t* represents the time interval ranging from 1 to *k*, as indicated in Equation (11).

(10) $$\mathrm{RMSE}=\sqrt{\frac{1}{n}\sum_{t=1}^{n}{\left(R_{t(k)-}-R_{t(k)}\right)}^{2}}$$

#### 3.4.2. Round Trip Time Analysis

The analysis of round-trip time (RTT) in energy trading tasks examines the reaction time of the tasks inside the energy trading architecture that is based on IoT orchestration. The assessment parameter is utilized to evaluate the capabilities of jobs performed within a digital twin-based IoT task orchestration system. The formula for RTT calculation is shown below:

(11) $$\mathrm{RTT}=t_{\mathrm{acknowledge}}-t_{\mathrm{send}}$$

where $t_{\mathrm{acknowledge}}$ is the time when the acknowledgment or completion notification is received, and $t_{\mathrm{send}}$ is the time when the task was initially sent.

#### 3.4.3. Latency Analysis

Latency analysis pertains to the assessment and enhancement of the time delay, commonly known as latency, encountered by tasks or jobs within a scheduling system. The process entails the examination of the duration required for tasks to initiate or conclude their execution from the point at which they are submitted or received within the system. The latency metric was utilized for the same objective as that of RTT. The formula for latency calculation is shown below, which comprises task start time, task submission time, task execution time, and task completion time.

(12) $$\mathrm{Latency}=T_{\mathrm{start}}-T_{\mathrm{submit}}+T_{\mathrm{execution}}+T_{\mathrm{completion}}$$

#### 3.4.4. Response Time Analysis

In the context of task orchestration, response time is the duration between submitting a task or request and receiving its completion response. It measures the overall efficiency and speed of the task orchestration system in responding to user-initiated actions.

(13) $$\mathrm{Response}\;\mathrm{Time}=T_{\mathrm{completion}}-T_{\mathrm{submit}}$$

In the end, this study compares the outcomes achieved by task scheduling by employing various scheduling strategies, including round robin, Qayyum et al. [17], and FEF [37].

## 4. Performance Analysis

This section provides a comprehensive summary of the implementation outcomes obtained for the architecture of nano-grid energy trading facilitated by the Internet of Things (IoT). The user presents details regarding the surplus energy held by a prosumer, followed by the identification of a residential entity interested in acquiring energy through a nano-grid. Table 3 presents a comprehensive overview of the technological instruments and programming languages utilized in the study being examined. Irradiance sensors have the ability to measure the magnitude of solar radiation received by photovoltaic (PV) panels. The available data can be employed to simulate the operational characteristics of solar panels under diverse weather conditions, as well as to optimize the positioning and orientation of the panels in order to maximize energy production. The sensor is affixed to the edge computing device referred to as the Raspberry Pi. The present study employs an analog irradiance sensor that is coupled to a Raspberry Pi edge device. The utilization of an analog to digital converter (ADC) facilitates the conversion of an analog signal into a digital signal, hence enabling the Raspberry Pi to detect and process the signal.

### 4.1. Prediction Module Outcome

**Figure 5:**
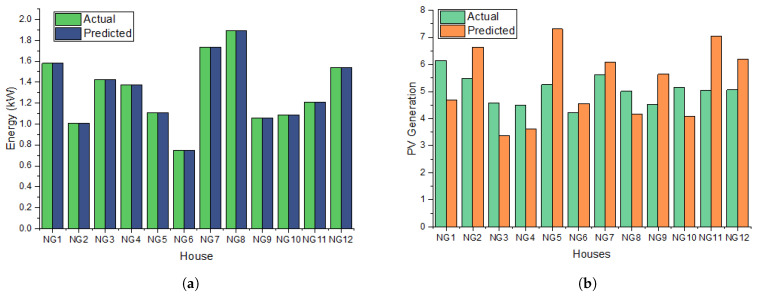

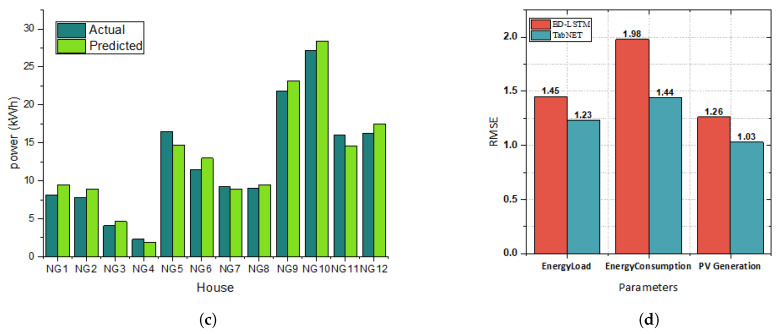
This figure shows actual and predicted values of key energy parameters, including actual and predicted energy load and photovoltaic (PV) generation values. (**a**) Actual and predicted energy values using gated recurrent unit (GRU) prediction module. (**b**) Predicted and actual PV generation values using GRU prediction module. (**c**) Predicted and actual energy consumption values using GRU prediction module. (**d**) Root mean square error value-based comparison analysis between Bidirectional-LSTM and GRU prediction module.

The employment of prediction models is of utmost importance in uncovering tacit knowledge and identifying concealed patterns within datasets. To facilitate the training of the model, the BD-LSTM approach integrates a 10-fold cross-validation methodology. This technique is employed to make predictions on various energy-related variables, namely energy load, energy consumption, PV generation, and energy cost. The process is visually depicted in Figure 5, specifically in sub-figures (a–c). The provided visual representations illustrate the observed and projected data pertaining to energy demand, photo-voltaic (PV) generation, and energy consumption, effectively emphasizing the discrepancies between the two datasets. In Figure 5d, it is evident that PV generation prediction exhibits the lowest root mean square error (RMSE) value of 1.03 among the considered parameters. Following this, the energy load reveals an RMSE of 1.23, while energy consumption exhibits the highest RMSE value of 1.44. In general, the obtained root mean square error (RMSE) value for the gated recurrent unit (GRU) model indicates a satisfactory degree of predictive accuracy. This outcome has important implications for energy-focused prediction models, as it enables energy provider organizations to make educated decisions based on reliable forecasts.

**Figure 6:**
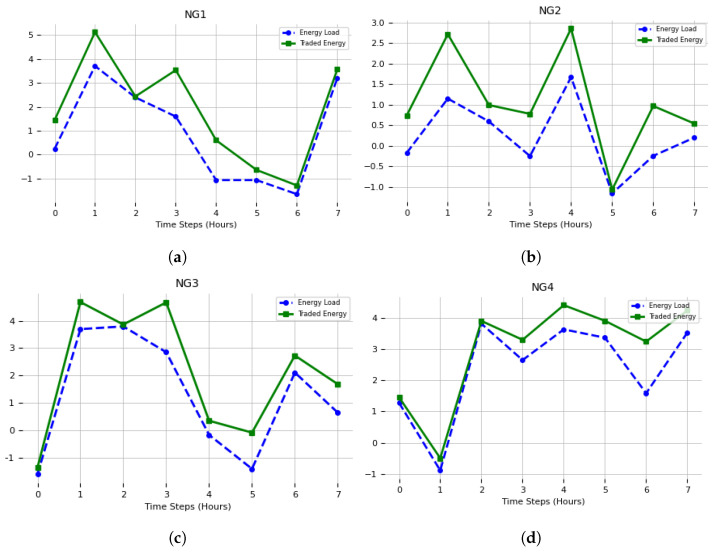

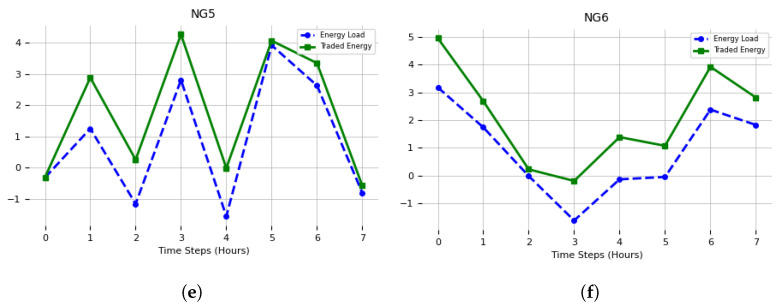
Energy load and traded energy values using energy storage system (ESS) power optimization for NG1 to NG6 (**a**–**f**).

The outcomes obtained from the recommendation model based on GRU are contrasted with a comparable investigation that employed BD-LSTM for predicting the same variables, as depicted in Figure 6. In general, a reduction in the root mean square error (RMSE) signifies the enhanced performance of the model, suggesting that the model’s predictions are in closer agreement with the observed values. Figure 6d illustrates that the root mean square error (RMSE) value achieved by the GRU prediction model is superior to the outcome of the BD-LSTM model. This suggests that the GRU model can be regarded as a more effective metric.

### 4.2. ESS Power Optimization

**Figure 7:**
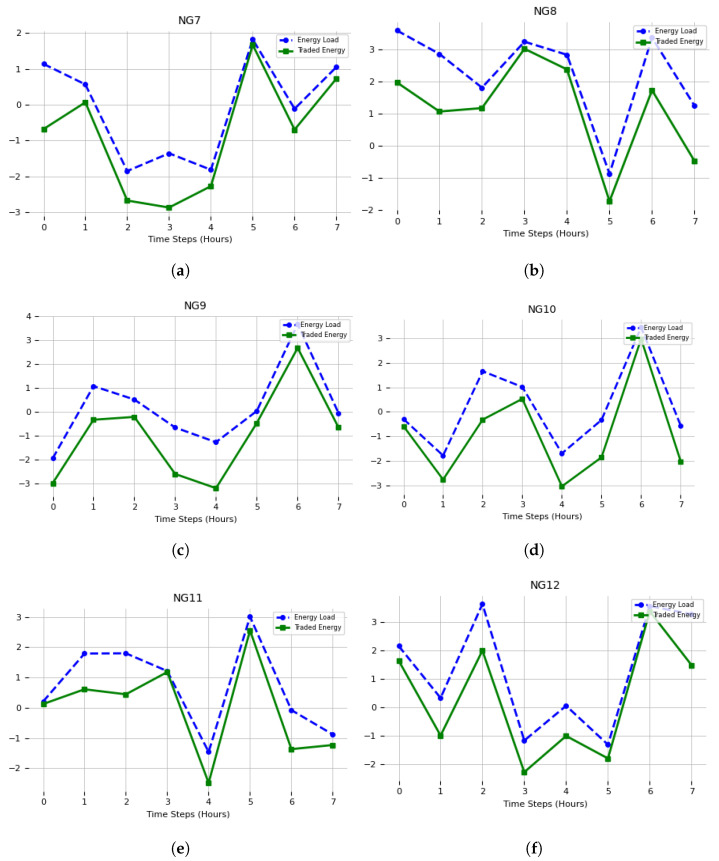
Energy load and traded energy values using energy storage system power optimization for NG7 to NG12 (**a**–**f**).

The next section presents the simulation outcomes for 12 of the total 21 nano-grids. The results are illustrated in Figure 6 and Figure 7, wherein the simulation outcomes are partitioned into two distinct sections. The figures presented depict the X-axis as representing the “Days of the Week”, and the Y-axis as representing “Power (kW)”. Positive numbers imply that a nano-grid functioned as a prosumer, engaging in energy trading by supplying excess energy to other interconnected nano-grids inside the network. In contrast, negative values signify that a nano-grid functioned as an energy consumer, obtaining energy from other nano-grids. The simulation reveals that each nano-grid assumes a distinct role. For instance, NG-10 undertook the position of a consumer due to its insufficient photovoltaic energy generation in meeting its energy requirements. In contrast, nano-grids 1, 2, and 4 exhibited prosumer behavior due to their energy consumption being lower than their photovoltaic energy production. As a result, the surplus energy was effectively employed for commercial transactions with other consumer nano-grids experiencing a demand for electricity. Moreover, the nano-grids that remained exhibited diverse behavior during the energy trading process, which was contingent upon their respective energy production capabilities. In cases where a nano-grid possesses surplus photovoltaic (PV) energy, it assumes the role of a prosumer, and in situations where it experiences a deficit of energy, it operates as a consumer nano-grid. The aforementioned results collectively indicate the successful execution of the suggested methodology for interconnected small-scale nano-grids functioning as a network.

On the other hand, nano-grids 1, 2, and 4 exhibited prosumer behavior since their energy generation from photovoltaic sources exceeded their energy consumption requirements. As a result, they effectively harnessed the excess energy to fulfill the energy requirements of additional consumer nano-grids through the process of energy sales. Moreover, the remaining nano-grids fulfilled diverse functions in the process of energy sharing, with each nano-grid’s role being defined exclusively by the amount of energy it created. To provide an illustration, in cases where a nano-grid possessed surplus photovoltaic (PV) energy, it operated as a prosumer, but in situations where its PV energy was inadequate, it operated as a consumer nano-grid.

**Figure 8:**
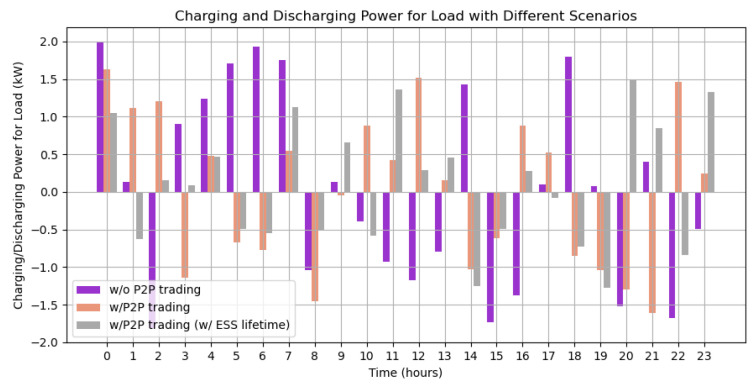
Average charging and discharging power of nano-grid houses based on Average charging and discharging power of nano-grid houses based on optimized energy storage system [19].

Similar to [19], we also computed average charging and discharging load values for particpating nanogrid houses. Figure 8 denotes the average charging and discharging values for load for all the participating 12 nano-grid houses for times-stamps of 24 h. It can be seen that when energy trading was processed without P2P trading, the load values were quite higher, whereas the best values are reported for P2P trading with optimized ESS values.

### 4.3. IoT Orchestration-Based Virtualized Energy Trading Outcomes

As previously stated, while examining the attributes of smart grid-based systems, the tasks can be classified into two categories: event-driven tasks and periodic tasks. The graphic illustrates the round-trip time analysis for a set of 10 nano-grid energy trading operations. Users have the ability to assign various tags to items based on their significance, such as sensor, light, or load. Users have the ability to give several types of tags to virtual objects. Subsequently, several techniques are employed to manipulate actuators, such as acquiring light and adjusting light intensity. The characteristics include sensing and actuating data. When the user selects the option “Add virtual objects”, the metadata associated with the virtual objects is stored in the virtual objects’ repository.

The horizontal axis of the graph depicts the number of smart houses, whilst the vertical axis portrays the evaluation of throughput in relation to the number of energy tasks performed per second. The x-axis represents the values of throughput in milliseconds, while the y-axis represents the number of households. As previously stated, the assessment comprises a total of 24 residences, which are categorized into three groups of 8, 16, and 24, as depicted in the diagram.

**Figure 9:**
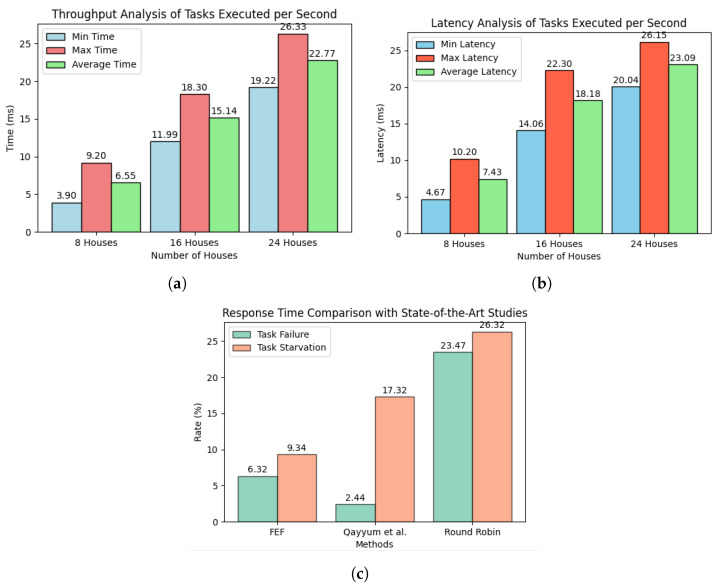
The provided figure outlines the assessment criteria employed to analyze the results of Internet if Things (IoT)-driven task orchestration in the context of nano-grid energy operations. This assessment comprises two key components: (**a**) An examination of throughput within the energy trading sector, encompassing tasks involved in IoT task orchestration. (**b**) An assessment of task latency during the execution of energy trading within nano-grids. (**c**) An inquiry into the comparison of task failure and task scarcity in IoT task orchestration, with a specific emphasis on cutting-edge scheduling strategies for executed tasks.

The analysis shown in Figure 9a depicts the throughput analysis of tasks executed per second for different numbers of houses in a nano-grid. Three sets of bars represent the minimum, maximum, and average times in milliseconds. As the number of houses increases from 8 to 24, there is a corresponding increase in the minimum, maximum, and average times. The minimum time ranges from 3.9 ms to 19.22 ms, the maximum time ranges from 9.2 ms to 26.33 ms, and the average time falls between these values. The chart provides a clear visual representation of the performance characteristics, highlighting the variability in task execution times across different house configurations in the nano-grid.

The bar chart shown in Figure 9b illustrates latency analysis for tasks executed per second in a nano-grid, comparing the minimum, maximum, and average latencies across different numbers of houses (8, 16, and 24). As the number of houses increases, there is a corresponding rise in both minimum and maximum latencies, providing insight into the performance characteristics of the system under varying workloads.

Similarly, the bar graph shown in Figure 9c compares task failure and task starvation rates across different scheduling methods—FEF, Qayyum et al., and Round Robin—expressed as percentages (0–25%). For task failure rates, Qayyum et al. exhibits the lowest at 2.44%, followed by FEF at 6.32%, and Round Robin at 23.47%. Conversely, in task starvation rates, FEF shows the lowest at 9.34%, followed by Qayyum et al. at 17.32%, and Round Robin at 26.32%. This visualization provides a succinct comparison of response time metrics for each method, aiding in the assessment of their efficiency and reliability in task execution.

The proposed methods demonstrate substantial improvements compared to existing studies. For task starvation, the proposed Round Robin (RR) and Time-Aware strategies achieved 13.8% and 17.4%, respectively, notably lower than the existing values of 27.5% and 22.3%. In task failure, the proposed RR and FEF strategies recorded 14.4% and 20.8%, surpassing the existing values of 38.7% and 36.5%. These results indicate the superiority of the proposed approaches in minimizing both task starvation and failure rates, showcasing enhanced system reliability and efficiency.

**Figure 10:**
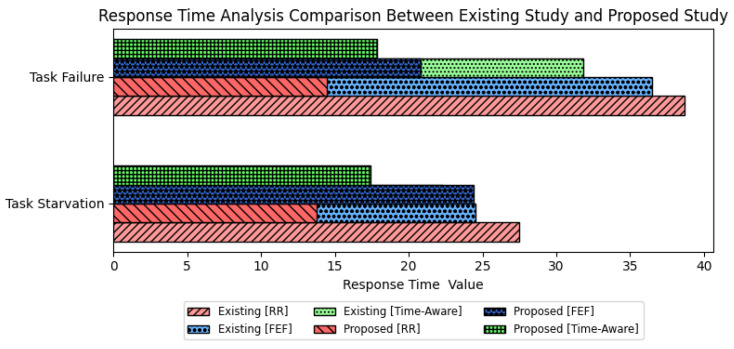
Comparison analysis for response time for real-time energy tasks executed in the proposed Internet of Things (IoT)-orchestrated energy trading system using three scheduling techniques.

A comparison is conducted with a prior study [17] to address the disparities in the dataset resulting from the restricted accessibility of comparable energy-related apps employing IoT task orchestration. The round-robin and FEF techniques are of considerable importance since they demonstrate the effectiveness of the strategy in minimizing latency for nano-grid residences. Figure 10 illustrates a comparative examination of response time in the present study and previous work. The analysis specifically concentrates on the occurrence of task starvation and task failure, employing round-robin, FEF, and time-aware scheduling techniques. In the present investigation, it is noteworthy that the round-robin and FEF algorithms exhibited a substantial reduction in occurrences of “task starvation” and “task failure”, indicating an enhancement in the efficiency and reliability of task execution.

### 4.4. Issues and Challenges

The implementation of the suggested study inside real-world energy trading situations may provide a number of obstacles. The expansion of the framework to accommodate a larger number of nano-grid dwellings presents a possible challenge in terms of scalability. This is due to the increasing computing requirements and the possibility of network congestion. The deployment of IoT devices, such as sensors and Raspberry Pi devices, poses hurdles in terms of hardware needs. These obstacles encompass the initial expenses associated with acquiring and implementing these devices, as well as compatibility issues that arise in locations with obsolete infrastructure. The process of incorporating the framework into pre-existing energy systems may encounter obstacles, such as challenges related to compatibility with outdated systems and opposition from conventional energy suppliers due to aversion towards change. The transmission of sensitive information gives rise to issues regarding data security and privacy, hence mandating the implementation of rigorous safeguards to prevent breaches and assure adherence to regulatory requirements. The establishment of a robust and dependable infrastructure for Internet of Things (IoT) devices is of paramount importance in order to achieve the desired outcomes. This necessitates the implementation of various strategies aimed at mitigating potential issues, such as malfunctions, technological problems, and power supply disruptions. The process of overcoming regulatory and legal obstacles in peer-to-peer energy trading entails traversing intricate frameworks and acquiring the required approvals. These challenges encompass resolving issues pertaining to data ownership, responsibility, and contractual agreements. The obstacles to obtaining cooperation from inhabitants lie in user acceptance and behavior, as there is a possibility of encountering opposition when introducing new technologies or attempting to modify energy usage habits. It is imperative to approach these difficulties in a comprehensive manner in order to effectively execute the framework in practical situations. This entails taking into account technological, regulatory, and sociological factors to guarantee the durability and efficacy of the framework.

## 5. Conclusions

In summary, this research study introduces a nano-grid energy trading system that utilizes IoT task orchestration and digital twin-based technologies to improve the effectiveness of conventional peer-to-peer trading methods. The solution that has been created integrates a task orchestration framework enabled by the Internet of Things (IoT). This framework automatically generates energy tasks by analyzing the service information provided by users. Subsequently, the aforementioned tasks are scheduled, taking into account temporal limitations, and dynamically assigned to tangible resources inside the proposed system. The optimization module enhances the scheduling process by incorporating particle swarm optimization. The initial step of the suggested architecture involves the deployment of a peer-to-peer energy trading system for the nano-grid. This is then followed by the integration of pertinent tasks into a task list that is formed. Virtual objects are generated to represent actual resources, and tasks are assigned to these virtual objects in an independent manner. The tasks are organized in a systematic manner to ascertain the most efficient sequence of execution and allocated to suitable sensors, thereby dynamically deploying them to the available physical resources. The performance of the virtualization module is assessed by the utilization of established metrics, including round-trip time (RTT), throughput analysis, latency analysis, and response time. The evaluation findings, in conjunction with comparisons to current leading methodologies, unequivocally establish the superiority of the suggested procedure, particularly within the smart grid field. In general, this architectural design presents a viable and practical option for the implementation of real-time smart grid systems in industrial settings. It efficiently manages the allocation of energy trading activities, contributing to sustainability and efficiency. The proposed approach demonstrates clear advantages in the administration of these tasks, thereby offering a noteworthy addition to the subject.

## Figures and Tables

**Table 1:** Contemporary state-of-the-art analysis of contemporary digital twin based technologies.

Approach	Focus	Advantage	Disadvantage
Orchestration in Smart Grid [17]	Real-time power allocation and task automation	Enhanced IoT applications, improved response	Emphasis on dynamic features, complexity
MDMT-MOA Architecture [5]	IoT enterprise design with orchestration	Efficient task coordination	Specific to enterprise IoT applications
CPSaaS Architecture [4]	Task orchestration in smart urban environments	Mission-critical architecture, predictive analysis	Specific to urban and fire detection
Healthcare Monitoring with IoT Orchestration [30]	Patient health monitoring with optimized scheduling	Improved response time, reduced latency	Focus on healthcare applications
Task-Level Orchestration [5]	Automation of service provision	Increased flexibility, dynamic management	Specific to service-oriented architecture
Task Scheduling Optimization [16]	Efficient task scheduling in IoT systems	Avoid resource idleness, improved performance	Challenges in real-time scheduling
IoT Task Orchestration [15,16]	Energy management in nano-grids	Efficient energy management, data-driven approach	Limited scope in nano-grid context

**Table 2:** Description of terms.

Term	Description
H	Participating nano-grid houses
battery	ESS system installed in-house
k	Index of house
A	Self-adequate energy
EL	Energy load
Photovoltaic	Photovoltaic power
remained	Remaining power
surplus	Surplus energy owned by N
rel	Releasing power
saved	Energy saved in ESS
sold	Sold energy
distributed	Energy distributed by all nano-grids

**Table 3:** Tools and Techniques used in the proposed system.

Technology Name	Detail
OS	Windows 10 for PC Server, Raspbian for edge Raspberry Pi
Programming language	Python Flask, JavaScript, HTML, CSS
Libraries	Bootstrap 3, Jinja 3, JSplumb for mapping
Server	Flask server
Persistence	MySQL
Browser	Chrome and Firefox
Core programming language	Python 3

## Data Availability

Data were collected by the authors and can be obtained upon request from the corresponding author.
